# Broadband electronic resonance coherent anti-Stokes/Stokes Raman scattering microscopy

**DOI:** 10.1126/sciadv.aec4772

**Published:** 2026-07-31

**Authors:** Yusuke Murakami, Minori Masaki, Norihide Sagami, Ikuto Eshima, Ryosuke Oketani, Masashi Yanagisawa, Hideaki Kano, Sakiko Honjoh, Kotaro Hiramatsu

**Affiliations:** ^1^ International Institute for Integrative Sleep Medicine (WPI-IIIS), Tsukuba Institute for Advanced Research (TIAR), University of Tsukuba, Ibaraki 305-8575, Japan.; ^2^ Ph.D. Program in Humanics, University of Tsukuba, Ibaraki 305-8577, Japan.; ^3^ Department of Chemistry, Faculty of Science, Kyushu University, Fukuoka 819-0395, Japan.; ^4^ Department of Molecular Genetics, University of Texas Southwestern Medical Center, Dallas, TX 75390, USA.; ^5^ Department of Biosciences and Informatics, Faculty of Science and Technology, Keio University, Kanagawa 223-8522, Japan.

## Abstract

Coherent Raman imaging (CRI) enables label-free chemical imaging based on intrinsic molecular vibrations, but its applicability is often limited by low sensitivity, hindering the detection of low-abundance biomolecules. Although electronic resonance enhances sensitivity, most resonance CRI implementations rely on narrowband excitation and/or detection, which limits spectral coverage and complicates the differentiation of target molecules from complex backgrounds. Here, we address these challenges by developing broadband electronic resonance coherent anti-Stokes/Stokes Raman scattering (BER-CARS/CSRS) microscopy. We show that BER-CARS/CSRS enables highly sensitive, label-free imaging of endogenous chromophores with broad spectral coverage of the entire fingerprint region. Specifically, we captured time-lapse images, visualizing low-abundance cytochromes alongside abundant biomolecules (lipids, proteins, and nucleic acids) in living human embryonic kidney (HEK) 293 cells. Furthermore, we applied the method to mouse tissues, highlighting characteristic localizations of cytochromes in the brain cortex, the cerebral ventricle wall, and the liver. Our results demonstrate that BER-CARS/CSRS provides highly sensitive, label-free chemical imaging from organelle dynamics to tissue mapping, enabling quantitative phenotyping and slide-scale histopathology.

## Teaser

Coherent Raman scattering enables label-free cytochrome imaging from live cell organelles to brain and liver tissues.

## INTRODUCTION

Coherent Raman imaging (CRI) is a powerful optical modality that enables label-free visualization of biomolecules in living cells and tissues by detecting their intrinsic vibrational frequencies. In CRI, molecular vibrations are coherently excited by nonlinear interactions induced by two-color laser fields, producing coherent optical signals that can be recorded with high spatial and temporal resolution. Representative implementations include coherent anti-Stokes Raman scattering (CARS) and stimulated Raman scattering (SRS) (*1*–*5*), which offer rapid image acquisition, three-dimensional sectioning capabilities, and molecular specificity without the need for fluorescent labels. These techniques have been applied across diverse contexts, such as in vivo monitoring of lipid droplets (*6*, *7*), nucleic acids (*2*, *8*–*10*), ex vivo visualization of amyloid plaques (*11*) and brain tumors (*12*), identification of microplastics in zooplankton (*13*), label-free histopathological diagnosis of metabolic diseases (*14*), gene-correlated metabolic imaging (*15*), and in vivo tracking of drug delivery carriers (*16*). Despite these advantages, the sensitivity of CRI techniques is typically limited to the millimolar range due to the inherently small Raman scattering cross section, making it challenging to visualize low-abundance molecules.

To overcome the sensitivity limit in CRI, harnessing electronic resonance is a promising approach. In electronic resonance Raman processes, the excitation wavelength is tuned to the absorption band of the target molecules, resulting in a marked increase in the Raman scattering cross section for vibrational modes coupled to the electronic transition (*17*–*19*). In spontaneous Raman imaging, resonance Raman enhancement has been widely used to visualize light-absorbing biomolecules such as carotenoids and cytochromes (*20*–*22*). Although electronic resonance CRI (*23*–*26*) has proven to be a promising approach due to its high sensitivity down to sub-micromolar level, its full potential has not yet been unlocked because most implementations rely on narrowband excitation and detection. Specifically, narrowband-detection CRI has two major drawbacks. First, molecular fingerprinting ability of Raman measurements is compromised, resulting in an ambiguous interpretation of the obtained Raman images. Second, in coherent Raman processes especially, vibrationally resonant signals are frequently obscured by nonspecific background from four-wave mixing or cross-phase modulation. In these cases, broadband detection is crucial for resolving overlapping peaks and enabling quantitative interpretation of the spectra.

In this work, we address these limitations by developing a broadband electronic resonance coherent anti-Stokes/Stokes Raman scattering (BER-CARS/CSRS) microscopy platform. Our system uses a near-infrared (NIR) supercontinuum (SC) source as the pump and Stokes fields, combined with a narrowband visible laser as the probe field, enabling broadband spectral acquisition across the fingerprint region under strong electronic resonance conditions. This configuration integrates the high sensitivity of electronic resonance with the wide spectral coverage essential for comprehensive molecular fingerprinting. Using BER-CARS/CSRS microscopy, we achieved label-free detection and imaging of endogenous light-absorbing biomolecules, including cytochromes in living human embryonic kidney (HEK) 293 cells as well as in mouse brain slices and liver tissues. These results highlight the capability of our method to provide quantitative, chemically specific imaging for a broad range of applications in life sciences and biomedical research.

## RESULTS

**Fig. 1.**
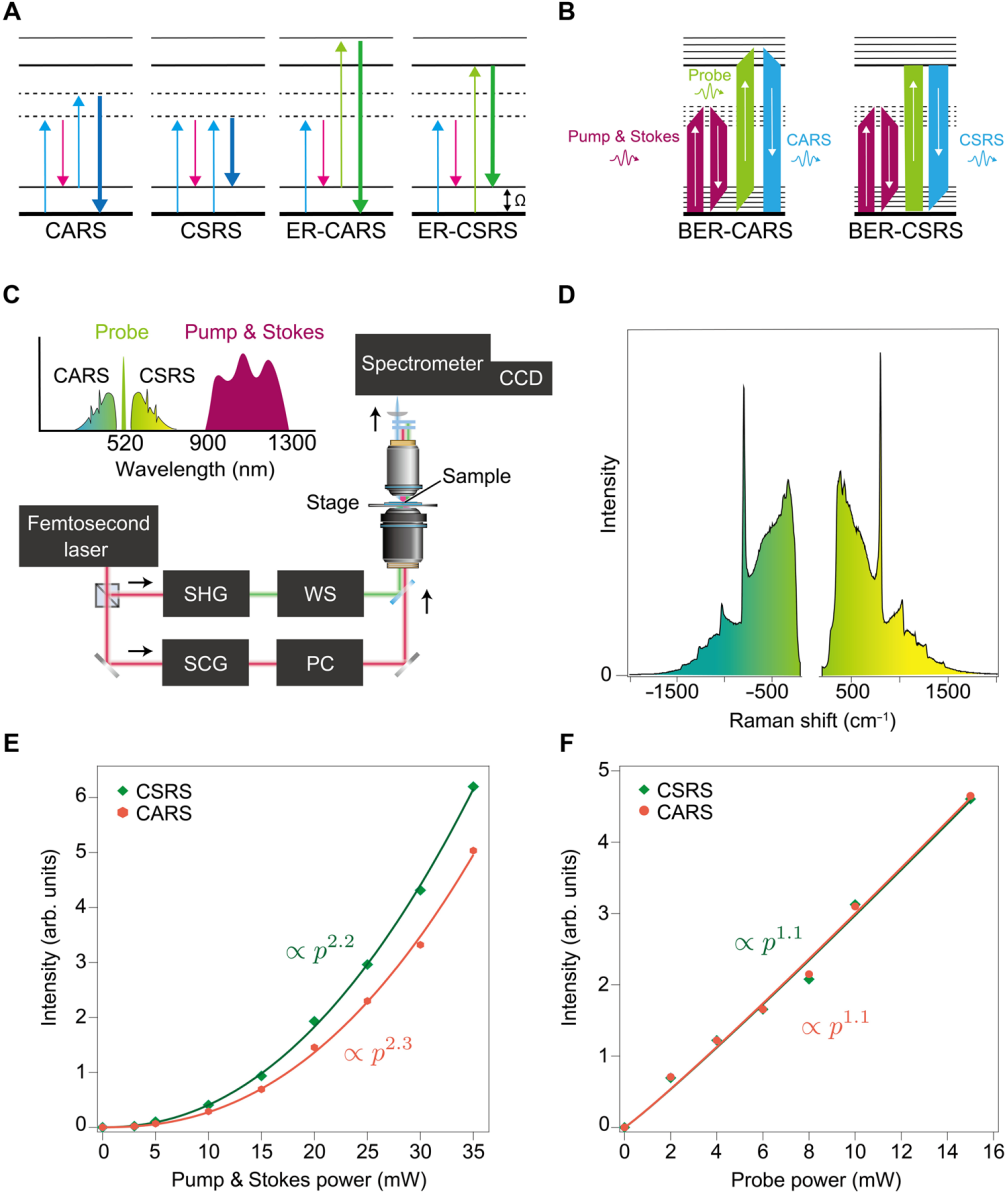
Concept and working principle of BER-CARS/CSRS microscopy. (**A**) Energy diagrams of CARS/CSRS and ER-CARS/CSRS processes. (**B**) Energy diagrams of the BER-CARS/CSRS processes. (**C**) Schematic of our BER-CARS/CSRS setup. Inset shows spectral profiles of the incident fields and the generated CARS and CSRS fields. SHG, Second-harmonic generation; WS, wavelength selector; SCG, SC generation; PC, pulse compressor. (**D**) Raw BER-CARS/CSRS spectrum of liquid cyclohexane. (**E**) Power dependence of BER-CARS/CSRS signals on the pump and Stokes intensity. arb. units, arbitrary units. (**F**) Power dependence of BER-CARS/CSRS signals on the probe intensity.

CARS and CSRS are third-order nonlinear optical processes induced by the pump, Stokes, and probe fields (Fig. 1A). In these processes, the pump and Stokes fields create vibrational coherences of the molecules, which subsequently interact with the probe field to generate a third-order nonlinear polarization that radiates CARS or CSRS fields. In electronic resonance CARS (ER-CARS) or electronic resonance CSRS (ER-CSRS), the photon energy is tuned to match an electronic transition energy of the molecule, resulting in resonance enhancement of the third-order nonlinear susceptibility χ^(3)^ and a substantial enhancement in signal intensity (Fig. 1A). To enable both broadband spectral acquisition and visible electronic enhancement, BER-CARS and BER-CSRS use a broadband pump and Stokes pulses in the NIR region (900 to 1300 nm) and a narrowband probe pulse in the visible region (520 nm) (Fig. 1B). The schematic of our developed BER-CARS/CSRS imaging system is shown in Fig. 1C (see the Materials and Methods section and fig. S1 for details). All the pulses were derived from a single femtosecond laser. To obtain the broadband pump ($\omega_{1}$) and Stokes ($\omega_{2}$) pulses, a portion of the laser output was coupled into a photonic crystal fiber (PCF) to generate an NIR femtosecond SC spanning from 900 to 1300 nm, which was subsequently compressed using a chirped mirror pair and a prism compressor. The narrowband probe ($\omega_{3}$) pulse was obtained by second-harmonic generation (SHG) using a nonlinear optical medium and wavelength selected with a 4-f filter. The two pulses were temporally and spatially overlapped using an optical delay line and a wavelength filter and guided into a custom-built microscope. The generated CARS and CSRS signals were spectrally filtered with a notch filter and a short-pass filter before being detected using a homebuilt spectrometer and a charge-coupled device (CCD) camera. Figure 1D shows a BER-CARS/CSRS spectrum obtained from liquid cyclohexane with a 1-ms exposure time, displaying symmetric spectral profiles on both the Stokes and anti-Stokes sides and vibrational peaks consistent with reported Raman spectra of cyclohexane. The input power dependence of the CARS/CSRS signal intensities (Fig. 1, E and F) shows a quadratic dependence on the pump/Stokes (NIR broadband) power and a linear dependence on the probe (visible narrowband) power. These dependences are consistent with the assumption that the CARS/CSRS signals were generated in the optical processes shown in Fig. 1B.

**Fig. 2.**
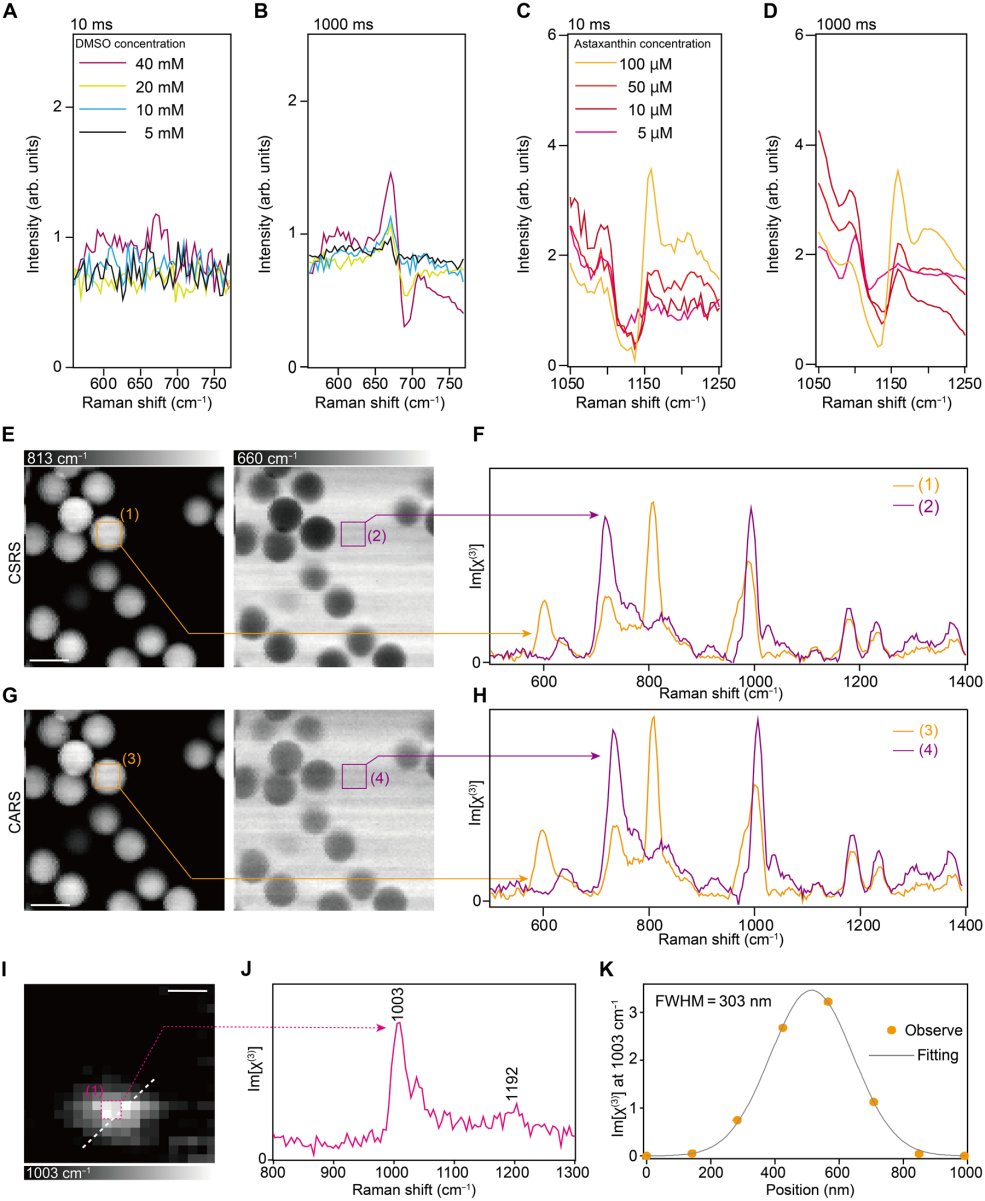
Concentration dependence and imaging performance characterization. (**A** and **B**) CARS spectra of DMSO solutions at concentrations of 40, 20, 10, and 5 mM at exposure times of (A) 10 ms and (B) 1000 ms. (**C** and **D**) CARS spectra of astaxanthin-acetone solutions at concentrations of 100, 50, 10, and 5 μM at exposure times of (C) 10 ms and (D) 1000 ms. (**E**) BER-CSRS images of PMMA beads at 813 and 660 cm^−1^. (**F**) Average Im[χ^(3)^] spectra from regions (1) and (2) in (E). (**G**) BER-CARS images of PMMA beads at 813 and 660 cm^−1^. (**H**) Average Im[χ^(3)^] spectra from regions (3) and (4) in (G). Image size, 101 by 101 pixels; pixel size, 0.5 μm by 0.5 μm; exposure time, 20 ms/pixel. Scale bar, 10 μm. (**I**) BER-CARS image of a 200-nm PS bead at 1003 cm^−1^. Image size, 20 by 20 pixels; pixel size, 100 nm by 100 nm; exposure time, 50 ms/pixel. Scale bar, 400 nm. (**J**) Average Im[χ^(3)^] spectra from the boxed region in (I). (**K**) Intensity profile along the dashed line in (I). Orange dots indicate observed values, and the gray curve indicates Gaussian fitting.

To evaluate the basic performance of our BER-CARS/CSRS imaging system, we first performed the concentration dependence study with water solutions of dimethyl sulfoxide (DMSO) and acetone solutions of astaxanthin at various concentrations. In this study, we focused on the CARS side of the spectra because clearer vibrational peaks of astaxanthin were detected in CARS compared to in CSRS due to lower fluorescence hindrance and higher resonance enhancement in the anti-Stokes region ($\lambda_{\text{max}}=477$ nm for astaxanthin). Figure 2 (A and B) shows CARS spectra of DMSO-water solutions, where the distinct peak at 670 cm^−1^ is clearly visible at 40 mM (10 ms) and 5 mM (1000 ms). Figure 2 (C and D) shows CARS spectra of astaxanthin-acetone solutions, where the distinct peak at 1152 cm^−1^ is visible at 10 μM in both exposure times. Because of the resonance enhancement, comparable signal intensities were obtained from astaxanthin at concentrations three to four orders of magnitude lower than those required for DMSO under the same exposure time and the excitation power. The limit of detection in BER-CARS in 10-ms exposure, which is a typical pixel dwell time in CARS imaging, is roughly 40 mM for DMSO (without electronic resonance) and 10 μM for astaxanthin (with electronic resonance). These results show that the resonance enhancement under visible excitation improves the detection limits of label-free imaging of visible-light-absorbing biomolecules.

We next evaluated the imaging performance of our setup using standard test samples. Polymethyl methacrylate (PMMA) beads with a diameter of 10 μm were suspended in immersion oil (Immoil-F30CC, Olympus) and imaged by raster scanning the sample using a translational stage (Fig. 2, E to H). For quantitative interpretation of the CARS/CSRS spectra, we extracted the imaginary part of χ^(3)^ by using the maximum entropy method (*27*). Image reconstruction was performed on the basis of the magnitude of Im[χ^(3)^] at 813 and 660 cm^−1^, where representative peaks of PMMA and the immersion oil are present, respectively. The CSRS (Fig. 2E) and CARS (Fig. 2G) exhibited similar profiles, which indicates that CARS and CSRS provide identical information in the nonresonant case. Figure 2 (F and H) shows average Im[χ^(3)^] spectra derived from CARS and CSRS measured in the regions identified by the rectangles in the images. The PMMA bead and the immersion medium are spectrally distinguished with rich chemical information in the entire fingerprint region.

We further assessed the capability of imaging nanoscale samples by imaging a 200-nm polystyrene (PS) bead. Figure 2I shows a CARS image reconstructed from Im[χ^(3)^] at 1003 cm^−1^, where a characteristic Raman peak of PS exists (Fig. 2J). This image indicates that our imaging platform resolves submicrometer structures. The Im[χ^(3)^] profile across a bead is shown in Fig. 2J, where Gaussian fitting analysis reveals that the full width at half maximum (FWHM) of the peak is approximately $d_{\text{obs}}=303\;\text{nm}$ (Fig. 2K). We estimated the FWHM of the point spread function by ${\left(d_{\text{obs}}^{2}-d^{2}\right)}^{1/2}=228\;\text{nm}$, where d=200 nm is the diameter of the bead. This value is larger than the theoretical estimation of 130 nm, estimated on the basis of the diffraction limit of the 520- and 1040-nm beams with third-order nonlinear interaction and an objective numerical aperture of 1.42. We think that this mismatch comes from imperfect spatial overlaps of the excitation beams, especially along the axial direction and nonideal beam profile of the output of the PCF.

**Fig. 3.**
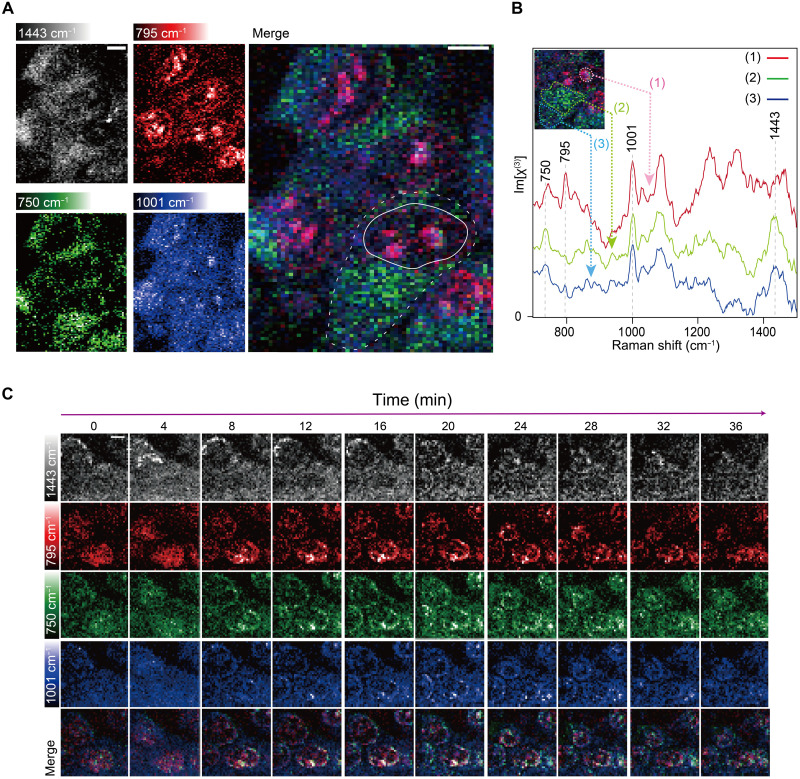
BER-CSRS imaging of living HEK293 cells. (**A**) BER-CSRS images of living HEK293 cells reconstructed at 1443 (lipids), 795 (nucleic acids), 750 (cytochromes), and 1001 cm^−1^ (proteins). Solid white line, nucleus; dashed white line, cell boundary. Image size, 61 by 101 pixels; pixel size, 0.5 μm by 0.5 μm; exposure time, 20 ms/pixel. Scale bars, 5 μm. (**B**) Average Im[χ^(3)^] spectra corresponding to regions (1) to (3) in the inset. The inset shows a magnified view of the cell in (A). (**C**) Time-lapse BER-CSRS imaging of HEK293 cells during apoptosis at 1443 (lipids), 795 (nucleic acids), 750 (cytochromes), and 1001 cm^−1^ (proteins). BER-CSRS imaging was initiated at *t* = 0 min and performed until *t* = 36 min with a 4-min interval. Image size, 51 by 51 pixels; pixel size, 0.5 μm by 0.5 μm; exposure time, 1 ms/pixel. Scale bar, 5 μm.

We applied BER-CARS/CSRS imaging to live HEK293 cells, a widely used human-derived cell line that serves as a convenient model for investigating fundamental cellular processes, including mitochondrial function (*28*, *29*). Cytochromes in these cells are central to energy metabolism (*30*) and apoptosis (*31*), yet their spatiotemporal dynamics, such as rapid redox changes and redistribution under stress, remain incompletely understood due to limited temporal resolution of conventional Raman approaches. Demonstrating that BER-CARS/CSRS can directly detect these chromophores in living cells thus represents a critical step toward elucidating their dynamic roles in cellular metabolism. Figure 3A shows BER-CSRS images of living HEK293 cells reconstructed by Im[χ^(3)^] at 1443, 795, 750, and 1001 cm^−1^, where the vibrational peaks of CH_2_ scissoring, pyrimidine ring/PO_2_^−^ backbone vibrations, heme breathing modes, and phenylalanine ring breathing are found, respectively. The spatial profiles in the images at 1443 and 1001 cm^−1^ primarily reflect the overall cell morphology, consistent with previously reported Raman images of lipids and proteins. In addition, the image at 795 cm^−1^ exhibited strong intensity in the nuclei and nucleoli, indicating that the signal at this frequency reflects the local abundance of nucleic acids. The image at 750 cm^−1^, where no clear contrast has previously been reported in conventional CRI bioimaging (*32*), shows high intensity at perinuclear regions. Considering the reported Raman peaks of cytochromes at 750 cm^−1^ and localization of cytochromes around the nuclei (*20*, *33*, *34*), these results suggest that our BER-CSRS platform successfully visualizes cytochromes in living cells. Figure 3B presents averaged Im[χ^(3)^] spectra from three regions indicated in the inset, enriched in nucleic acids (1), cytochromes (2), and proteins (3). Region (1) shows prominent peaks at 795 cm^−1^, (2) at 750 cm^−1^, and (3) at 1001 cm^−1^, further supporting the above assignment of the signals. Furthermore, simultaneous acquisition of BER-CARS and BER-CSRS images was performed to assess the performance of the two techniques, as shown in fig. S2. The signal-to-noise ratio (SNR) was estimated by calculating the peak height at the protein-rich region (indicated by the dashed line) divided by the SD in the background region. These results demonstrate that BER-CARS and BER-CSRS produce similar spatial contrasts and SNR (BER-CARS ≈ 6.9; BER-CSRS ≈ 5.2) in living HEK293 cells, underscoring the reliability of imaging across these two modalities.

We next performed time-lapse BER-CARS/CSRS imaging of HEK293 cells undergoing apoptosis (Fig. 3C and movie S1). HEK293 cells were incubated in Hanks’ balanced salt solution (HBSS) for 1 hour, mounted on a slide, with the observation starting at t=0 min. The reconstructed BER-CSRS images captured dynamic changes in lipids (1443 cm^−1^), nucleic acids (795 cm^−1^), cytochromes (750 cm^−1^), and proteins (1001 cm^−1^) in a cell at a frame rate of 0.25 frames/min. In the early stage, cytochromes were uniformly distributed within the nucleus (Fig. 3C, t=0 to 4 min). This is consistent with early apoptotic nuclear translocation of cytochrome c, implicated in chromatin condensation and caspase activation (*35*). Actually, the BER-CSRS image at 795 cm^−1^ taken at t=8 min (Fig. 3C) shows spatial patterns indicative of chromatin condensation. Nuclear cytochromes colocalize with nucleic acids inside the nucleus during apoptosis (Fig. 3C). These observations are consistent with the models in which cytochromes regulate chromatin during the DNA damage response (*35*, *36*) and provide label-free confirmation of this sequence without fluorescent probes. Furthermore, the BER-CARS/CSRS platform can achieve a frame rate of 2 frames/min using a rapid translational stage for sample positioning (fig. S3), suggesting the potential for observing even faster intracellular dynamics.

**Fig. 4.**
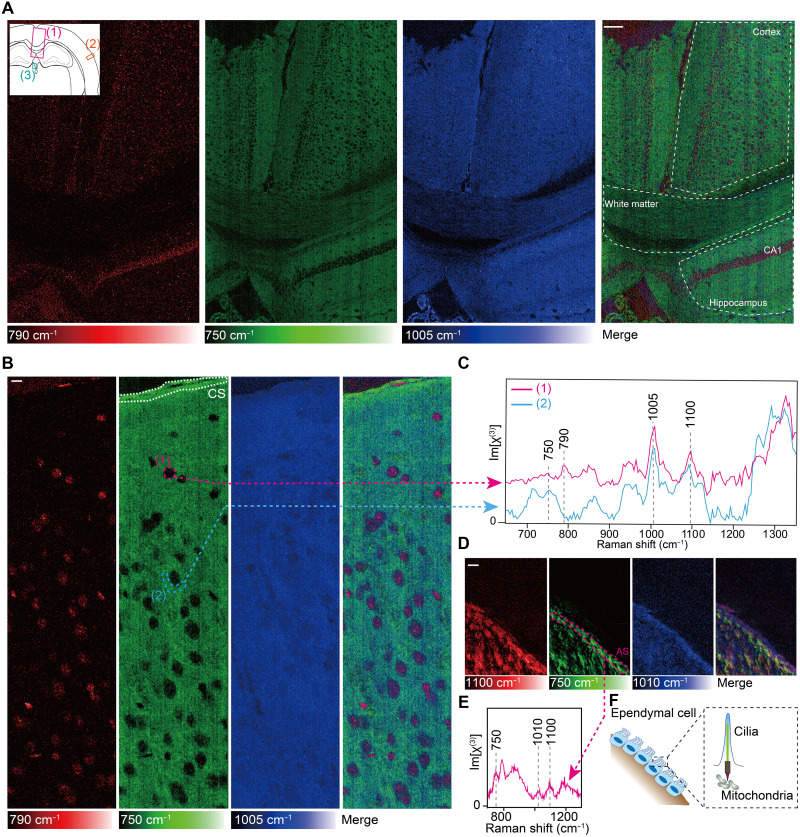
Chemical imaging of mouse brain tissue by BER-CARS microscopy. (**A**) Large-area BER-CARS imaging of the mouse brain across the cortex, white matter, and hippocampus. The region indicated as (1) in the inset was measured. Signals at 790, 750, and 1005 cm^−1^ correspond to nucleic acids, cytochromes, and proteins, respectively. Image size, 801 by 501 pixels; pixel size, 2 μm by 2 μm; exposure time, 5 ms/pixel. Scale bar, 100 μm. (**B**) High-resolution BER-CARS imaging of the cortex. The area measured at (2) in the inset of (A). CS, cortical surface. Image size, 801 by 189 pixels; pixel size, 0.5 μm by 0.5 μm; exposure time, 5 ms. Scale bar, 5 μm. (**C**) Average Im[χ^(3)^] spectra corresponding to regions (1) and (2) in (B). (**D**) High-resolution BER-CARS imaging around the cerebral ventricle wall. The area measured at (3) in the inset of (A). AS, apical surface. Signals at 1100, 750, and 1010 cm^−1^ correspond to nucleic acids, cytochromes, and proteins, respectively. Image size, 101 by 161 pixels; pixel size, 0.5 μm by 0.5 μm; exposure time, 5 ms/pixel. Scale bar, 10 μm. (**E**) Average Im[χ^(3)^] extracted from the region indicated by the magenta dotted line in (D). (**F**) Schematic illustration of the measurement region. Mitochondria are localized at the base of the cilia in ependymal cells.

To further showcase the utility of BER-CARS for visualizing complex tissue, we imaged mouse brain slices and leveraged electronic resonance enhancement to detect a cytochromes-associated heme band near 750 cm^−1^. Given that mitochondria govern neuronal energy supply and signaling, and because their remodeling modulates brain-state control, mapping of mitochondrial distribution provides biologically important insights (*37*–*39*). First, we acquired a large-area scan spanning the cerebral cortex, white matter, and hippocampus [Fig. 4A, region (1)]. Here, we used BER-CARS for constructing tissue images to avoid the autofluorescence background seen in CSRS. These macroscopic regions are readily distinguished by chemical contrast: The nucleic acid band at 790 cm^−1^ is enriched in cell body–dense cortical layers and the CA1 region, whereas the band at 1005 cm^−1^, attributed to proteins, appeared relatively uniform across the brain tissue. Also, cytochromes were visualized by image reconstruction at 750 cm^−1^, showing nonlocalized distribution within the cytoplasm. Broadband and electronic resonance capability of the present method enables multimodal chemical imaging including low-abundance molecules such as cytochromes at the tissue scale.

We next conducted higher-resolution imaging of the cortex [Fig. 4, A, region (2), B, and C], resolving chemical features at the single-cell level. The signal at 790 cm^−1^ localized to nuclei, sharply delineated from the cytoplasm, whereas the cytochrome-associated signal at ∼750 cm^−1^ and the protein-associated signal at 1005 cm^−1^ were broadly detected in the cytoplasm. Notably, the mean intensity of the band at ∼750 cm^−1^ is higher near the cortical surface (CS). A similar mitochondrial behavior has been observed in the fluorescence image of the mouse brain, where HK1 was stained and a higher fluorescence intensity was detected at the brain surfaces (*40*, *41*). Explicit discussion about the higher mitochondrial concentration at the surface was not provided in previous works presumably because higher fluorescence signals at the surface can be an artifact during immunostaining due to higher exposure to the fluorescence dye at the tissue peripheral. As CARS directly detects cytochromes without labeling, the higher mitochondrial concentration at the surface is now more evident. Although the underlying biological mechanisms need to be further investigated in future study, it may be related to high metabolic activity at the axon terminal (*42*, *43*), which necessitates mitochondria.

We performed BER-CARS imaging of the surrounding cerebral ventricle wall [Fig. 4, A, region (3), and D to F]. Ependymal cells, lining the cerebral ventricle wall, are known to have cilia for circulating cerebrospinal fluid, and their active motility is energetically supported by numerous mitochondria densely packed at the ciliary base (*44*). Figure 4D shows BER-CARS images of ependymal cells at 1100, 750, and 1010 cm^−1^, which correspond to the vibrational peaks of PO_2_^−^ stretching, heme breathing modes, and phenylalanine ring breathing, respectively. These peaks are attributed to nucleic acids, cytochromes, and proteins. In the BER-CARS image, we found localized cytochromes signals on the apical surface. The ability of our method to visualize mitochondrial cytochromes in ependymal cells can be an important tool to study ciliopathy because the lack of mitochondrial activity is one important origin of ciliopathies, such as diabetes, infertility, and obesity (*45*).

**Fig. 5.**
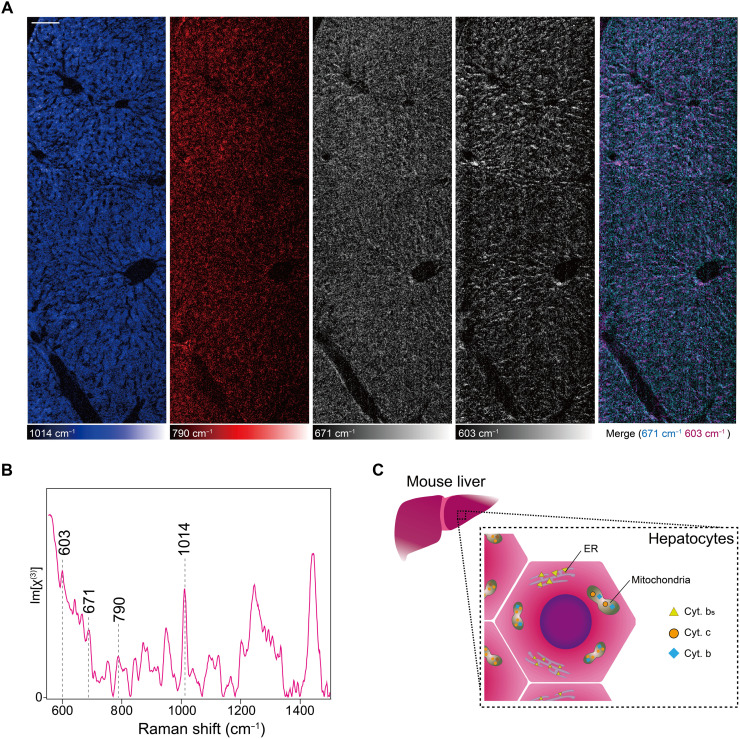
Spectroscopic imaging of cytochrome in mouse liver tissue using BER-CSRS microscopy. (**A**) BER-CSRS images of mouse liver tissue reconstructed at 1014 (proteins), 790 (nucleic acids), 671 (cytochrome b/b_5_), and 603 cm^−1^ (cytochrome c). Image size, 251 by 701 pixels; pixel size, 2 μm by 2 μm; exposure time, 20 ms. Scale bar, 50 μm. (**B**) Average Im[χ^(3)^] spectra of tissue. (**C**) Schematic illustration of the mouse liver. Cytochromes c and b are localized within mitochondria, whereas cytochrome b_5_ resides in the endoplasmic reticulum (ER).

Last, we applied BER-CSRS imaging to mouse liver tissue, a highly metabolically active organ, to visualize closely related cytochrome families. In hepatocytes, cytochrome c serves as a central electron carrier in the mitochondrial respiratory chain and plays a critical role in apoptosis regulation. In addition, cytochromes b and b_5_ respectively play important roles in the mitochondrial electron transport chain and endoplasmic reticulum–associated redox reactions including lipid metabolism (*46*) and drug detoxification (*47*). Distinctive measurement of these cytochrome families is essential for clarifying their respective roles in mitochondrial and endoplasmic reticulum–associated redox processes involved in hepatic metabolism and detoxification. Figure 5A shows BER-CSRS images of mouse liver tissue reconstructed from Im[χ^(3)^] at 1014, 790, 671, and 603 cm^−1^ (Fig. 5B). Images at 1014 cm^−1^ (proteins) and 790 cm^−1^ (nucleic acids) reflect the overall tissue structure and nuclear regions, respectively. Although the peak at 750 cm^−1^ is useful for the visualization of cytochromes due to its high intensity as shown in Figs. 3 and 4, it does not provide the specificity to differentiate cytochrome species. On the basis of previous studies (*48*, *49*), the Raman peaks at 603 and 671 cm^−1^ are assignable to cytochrome c and cytochrome b/b_5_, respectively. The images reconstructed from the Im[χ^(3)^] values at 603 and 671 cm^−1^ (Fig. 5A) show different spatial profiles; the image at 671 cm^−1^ shows delocalized distribution over the entire cytoplasm region, whereas that at 603 cm^−1^ shows higher contrast with partial overlap with the distribution at 671 cm^−1^. This observation is consistent with the well-established understanding of the intracellular distributions of cytochrome families, where cytochromes b and c are localized in mitochondria and cytochrome b_5_ predominantly exists in the endoplasmic reticulum (Fig. 5C) (*50*–*52*). These results demonstrate the ability of BER-CSRS to distinguish cytochrome species.

## DISCUSSION

We developed a BER-CARS/CSRS microscope that brings electronic resonance enhancement to CRI while retaining broadband vibrational fingerprinting. Although raw CARS/CSRS signals are generally more difficult to interpret quantitatively than SRS because of the nonresonant background contribution, by extracting the imaginary component of χ^(3)^, the platform provides quantitatively interpretable, background-mitigated spectra across the fingerprint region. These capabilities enabled label-free, time-lapse visualization of endogenous cytochromes (e.g., cytochrome c) in living cells and robust mapping of their distributions in complex mouse brain tissue and liver tissue. In particular, we observed spatial patterns ranging from mitochondria-associated structures to enriched signals at the apical surface of ependymal cells, consistent with known mitochondrial accumulation at this interface. Moreover, BER-CARS/CSRS enables us to distinctly visualize cytochrome c and cytochrome b/b_5_ in a label-free manner.

Although the developed BER-CARS/CSRS has unique imaging capabilities of various biomolecules, it is worth specifically comparing its cytochrome visualization capabilities with other methods: fluorescence imaging and spontaneous Raman imaging. Although fluorescence imaging enables highly sensitive and selective visualization of cytochromes, it requires staining or gene editing for making cytochromes fluorescent. This inherently perturbs the environment of the cytochromes molecules and makes it difficult to study their native behavior including their redox states. Spontaneous Raman imaging enables us to visualize and distinguish different cytochrome species such as reduced/oxidized cytochrome c/b/b_5_/P450 in a label-free manner (*48*, *49*). However, spontaneous Raman scattering is inherently weak, making it difficult to perform high-speed imaging. Compared to these methods, BER-CARS/CSRS realizes both high-speed capability and label-free detection of various cytochrome species although it requires a complicated setup using an ultrafast laser, potentially resulting in higher phototoxicity compared to the spontaneous Raman methods.

Besides the biological applications shown in the present work, our BER-CARS/CSRS imaging platform has the potential to elucidate various biological phenomena in cells and tissues. The combination of broadband coverage and resonance enhancement is well suited to tracking redox-dependent spectral changes of cytochromes, informing analyses of mitochondrial electron-transport activity, apoptotic signaling, and cellular responses to oxidative stress, as well as activity-dependent metabolism in neural circuits. The same principles extend to other key endogenous chromophores, including flavins, melanin, and hemoglobin, and can be leveraged to push the sensitivity of Raman tags toward highly specific, multiplexed molecular tracking.

Electronic resonance enhancement is known to introduce additional spectral distortion (*24*, *26*, *53*). Nevertheless, previous coherent Raman studies have suggested that both SRS and CARS signals remain effectively linear with concentration for endogenous chromophores at micromolar to millimolar concentrations (*23*, *54*). Therefore, BER-CARS/CSRS offers the sensitivity advantage of electronic resonance enhancement, with the potential to support quantitative imaging.

Although the present BER-CARS/CSRS microscope is already a powerful tool in biological research, its performance can further be enhanced in multiple directions. First, implementing epi-detection for both CARS and CSRS will enable measurements sensitive to the spatial frequency of the distribution of Raman-active molecules. This is because epi-detected CARS and CSRS show different object-frequencies dependence (*55*). Simultaneous forward/epi readout across CARS and CSRS therefore provides complementary sensitivity that helps evaluation of spatial inhomogeneity of the sample. Second, by generating broadband pump/Stokes light in the visible with sufficient difference-frequency coverage (∼580 to 670 nm), the system can interrogate the CH-stretch region (∼2800 to 3100 cm^−1^) concurrently with the fingerprint band, enabling imaging with richer chemical specificity. Third, coregistration with other nonlinear contrasts, including SHG, THG (third-harmonic generation), and two-photon excitation fluorescence, will yield a higher multimodality that fuses structural and morphological information with chemically specific BER-CARS/CSRS maps. Last, integration with information science (task-specific priority, physics-informed spectral unmixing, active acquisition, and anomaly detection) can drive more goal-oriented measurements, for instance, pathological triage, cell cycle staging, or discovery of atypical regions, reducing dwell time and dose while increasing diagnostic yield.

## MATERIALS AND METHODS

### Experimental setup for BER-CARS/CSRS microscopy

Our homebuilt BER-CARS/CSRS microscopy setup is illustrated in fig. S1. The light source was a femtosecond laser (FLINT FL2-SP, Light Conversion) operating at 1040 nm with a 50-fs pulse duration and a 76-MHz repetition rate. The output was split into two arms to generate NIR pump-Stokes pulses and a visible probe pulse. For the pump and Stokes beams, a fraction of the output was coupled into a PCF (LMA-PM-5, Thorlabs) to produce an SC. An automatic fiber alignment controller (KNA-VIS, Thorlabs) mounted on a three-axis stage (MAX313D, Thorlabs) was used for stable coupling and wavelength tuning. Group-delay dispersion introduced by the optics was compensated with chirped mirrors and a prism-pair compressor. For the probe beam, the remaining output was frequency doubled in a periodically poled lithium niobate (PPLN) crystal to 520 nm by SHG. The SHG beam was spectrally narrowed with a 4-f spectral filter (grating-lens pair, a slit, and a lens-grating pair), yielding a narrowband probe. A motorized delay line in the probe arm provided precise temporal overlap with the pump-Stokes pulses at the sample plane. The pump, Stokes, and probe beams were combined collinearly with a dichroic mirror and delivered to a laser-scanning microscope. The beams were focused onto the sample with an oil-immersion objective [UPLSAPO 60×/numerical aperture (NA) 1.42, Olympus], and the generated CARS and CSRS signals were collected with a second objective (Plan Apo 20×/NA 0.75, Nikon). The sample was mounted on a motorized linear stage (MLS203-2, Thorlabs) for scanning, whereas a piezoelectric stage (Nano-LPQ, Mad City Labs) was used for high-speed imaging (fig. S3). Excitation light was blocked with appropriate notch and long-pass filters, and the signals were dispersed by a custom-made spectrometer and detected with a CCD camera (PIXIS 100BR-DD eXcelon, Princeton Instruments). The imaginary part of χ^(3)^, which is equivalent to the spontaneous Raman spectrum, was retrieved from the raw CARS/CSRS data using the maximum entropy method (*27*). Im[χ^(3)^] spectra were preprocessed using the rolling-ball algorithm for background subtraction. Imaging data of HEK293 cells were analyzed by singular value decomposition (SVD) (Fig. 3A) and Tucker decomposition (Fig. 3C), where a difference Raman intensity at two wave numbers was mapped. For mouse brain and liver tissues (Figs. 4 and 5), intensity mapping was performed using amplitudes obtained from Gaussian fitting.

### Standard sample preparation

PMMA beads (10 μm in diameter; Polysciences Inc.) were suspended in immersion oil (Immoil-F30CC, Olympus), and PS beads (200 nm in diameter; Polysciences Inc.) were dispersed in water. Each sample was mounted between a coverslip and a microscope slide and sealed with nail polish.

### Cell culture

HEK293 and HeLa cells (RIKEN BRC Cell Bank) were cultured on coverslips in Dulbecco’s modified Eagle’s medium (DMEM; Fujifilm Wako) supplemented with 10% fetal bovine serum at 37°C in a humidified 5% CO_2_ atmosphere for 3 days. Before imaging, coverslips were rinsed in HBSS and incubated for 1 hour. The coverslip with adherent cells was then inverted onto a glass slide and sealed with nail polish.

### Animals and tissue preparation

C57BL/6N wild-type male mice were housed under controlled temperature and humidity on a 12-hour light/12-hour dark cycle. All animal experimental procedures were approved (approved protocol ID #24-324) and conducted following the guidelines established by the Institutional Animal Care and Use Committee of the University of Tsukuba. For liver tissue preparation, mice received rifampicin (RIF; 10 mg/kg, Tokyo Chemical Industry, #R0079) diluted in 5% DMSO/95% corn oil via intraperitoneal injection once daily for 3 consecutive days. On the fourth day, RIF-treated mice were deeply anesthetized with 5% isoflurane followed by intraperitoneal somnopentyl and transcardially perfused with phosphate-buffered saline (PBS), followed by 4% paraformaldehyde (PFA) in PBS. Livers were dissected and postfixed in 4% PFA at 4°C overnight, cryoprotected sequentially in 15% sucrose for 1 day and 30% sucrose for 1 day, embedded in OCT compound (Sakura Finetek), and frozen at −80°C. Livers were cryosectioned at 40 μm in thickness.

Separately, brain samples were obtained from untreated mice, which were subjected to the same anesthesia and perfusion procedures, followed by PFA fixation and cryosectioning. In the imaging experiments, data shown in Fig. 4 (A to C) were obtained from the same brain, whereas those in Fig. 4 (D and E) were obtained from different mice.
