# Supplementary material for: Broadband electronic resonance coherent anti-Stokes/Stokes Raman scattering microscopy
Source: Sci Adv. 2026 Jul 31;12(31):eaec4772. doi: 10.1126/sciadv.aec4772 (PMC13426426; doi:10.1126/sciadv.aec4772)
Supplement: Supplementary file 1 — Figs. S1 to S3 Legend for movie S1 Legend for data S1 [file sciadv.aec4772_sm.pdf]

Science Advances
AAAS

Supplementary Materials for

Broadband electronic resonance coherent anti-Stokes/Stokes Raman scattering microscopy

Yusuke Murakami *et al.*

Corresponding author: Kotaro Hiramatsu, hiramatsu@chem.kyushu-univ.jp

Sci. Adv. **12**, eaec4772 (2026)
DOI: 10.1126/sciadv.aec4772

The PDF file includes:

Figs. S1 to S3
Legend for movie S1
Legend for data S1

Other Supplementary Material for this manuscript includes the following:

Movie S1
Data S1

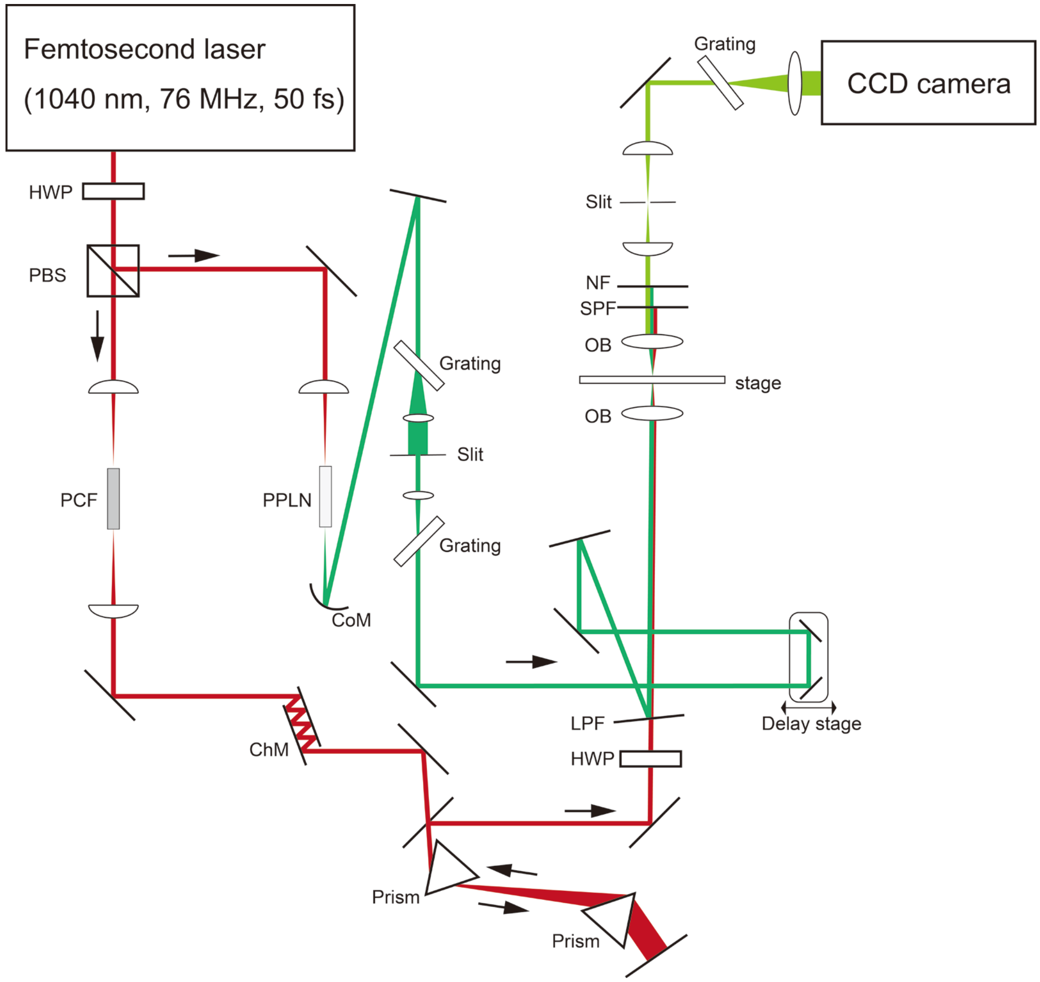

Fig. S1. Optical setup for BER-CARS/CSRS system. HWP: half-wave plate; PBS: polarizing beam splitter; PCF: photonic crystal fiber; PPLN: periodically poled lithium niobate; ChM: chirp mirror; CoM: concave mirror; LPF: long pass filter; OB: objective lens; SPF: short pass filter; NF: notch filter.

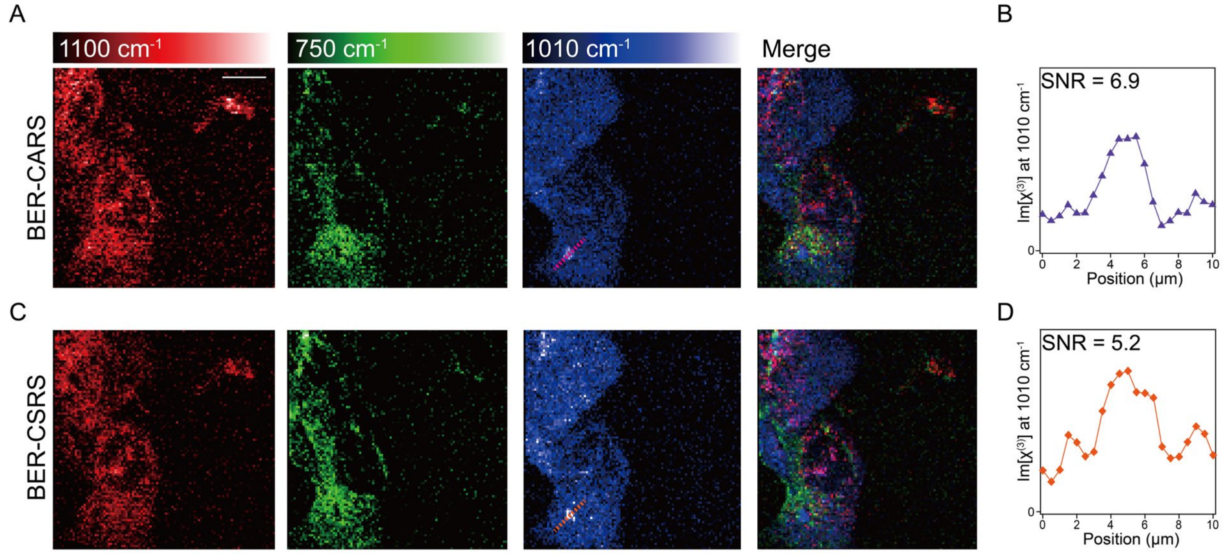

Fig. S2. Evaluation of the signal-to-noise ratio in BER-CARS and BER-CSRS imaging of HEK293 cells. (**A**) BER-CARS images of HEK293 cells at 1100 cm^{-1} (nucleic acids), 750 cm^{-1} (cytochromes) and 1010 cm^{-1} (proteins). (**B**) Intensity profile along the dashed line in the 1010 cm^{-1} image shown in (**A**). (**C**) BER-CSRS images of HEK293 cells at1100 cm^{-1} (nucleic acids), 750 cm^{-1} (cytochromes) and 1010 cm^{-1} (proteins). (**D**)Intensity profile along the dashed line in the 1010 cm^{-1} image shown in (**D**). The image size: 101 × 101 pixels, pixel size: 0.5 µm x 0.5 µm, exposure time: 20 ms/pixel, Scale bar: 10 µm. SNR: Signal to noise ratio.

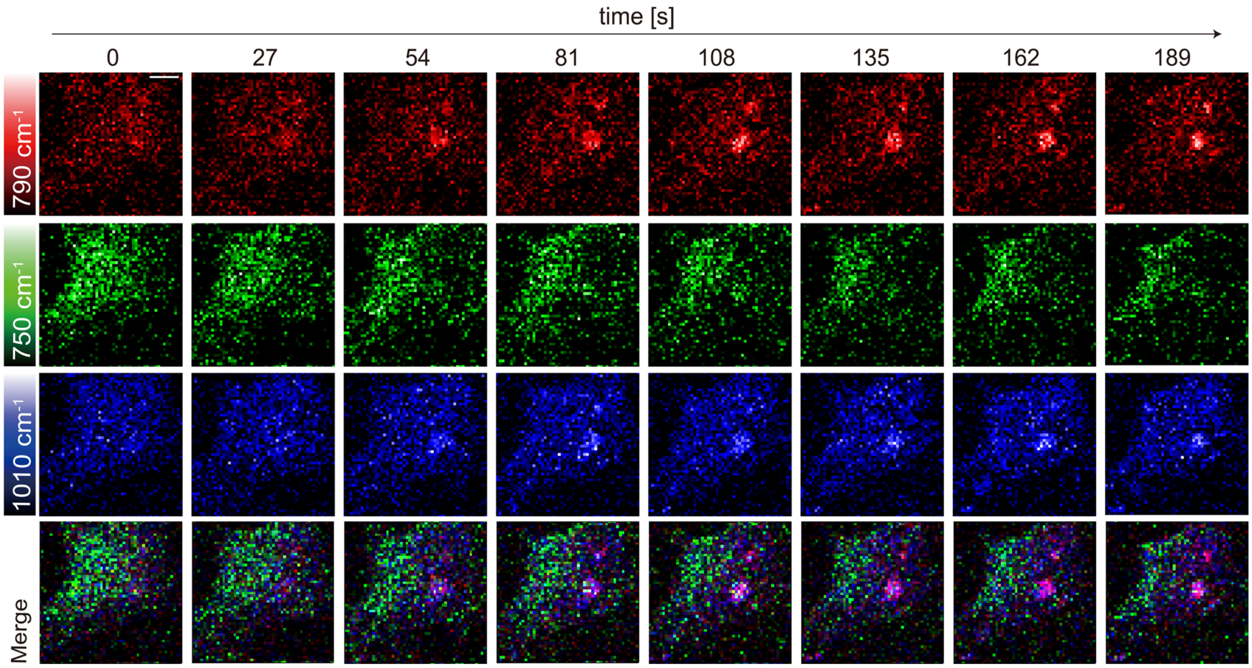

Fig. S3. High speed time lapse BER-CSRS imaging of HeLa cells at 790 (nucleic acids), 750 (cytochromes) and 1010 cm⁻¹ (proteins). BER-CSRS imaging was initiated at t = 0 s and performed until t = 189 s with a 27-s interval. The image size: 51 × 51 pixels, pixel size: 0.5 µm x 0.5 µm, exposure time: 1 ms/pixel, Scale bar: 5 µm.

Movie S1. Time-lapse BER-CSRS imaging of living HEK293 cells.

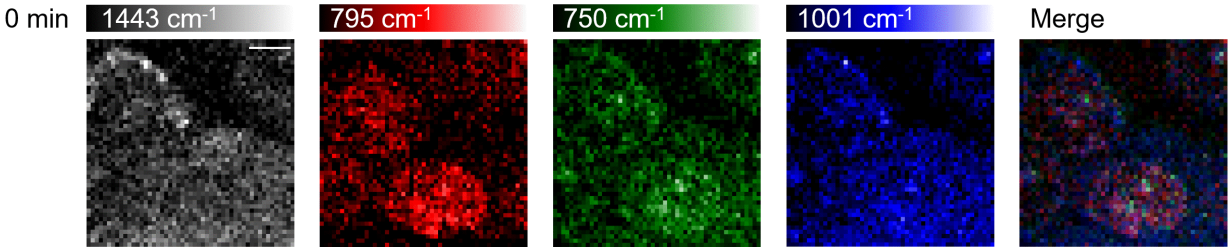

Data S1. Spectral and imaging numerical datasets for Figures 1 to S3.
